# Functional implications of atypical action potential generation in the (patho)physiological brain: from developmental program to glioma

**DOI:** 10.3389/fncir.2026.1851817

**Published:** 2026-06-09

**Authors:** Tong Tong, Anders Rosendal Korshøj, Wen-Hsien Hou

**Affiliations:** 1 Department of Health Science and Technology, Aalborg University, Aalborg, Denmark; 2 Department of Neurosurgery, Aarhus University Hospital, Aarhus, Denmark; 3 Department of Clinical Medicine, Aarhus University, Aarhus, Denmark; 4 Department of Biomedicine, Aarhus University, Aarhus, Denmark; 5 Department of Biomedicine, Center for Proteins in Memory (PROMEMO), Danish National Research Foundation, Aarhus University, Aarhus, Denmark

**Keywords:** atypical action potentials, glioma, intrinsic excitability, neurodevelopment, neuron–glioma interaction, oligodendrocyte precursor cells, patch-clamp, spikelets

## Abstract

Atypical action potentials (aAPs) are fast depolarizing electrical spikes recorded from the cell body, with a smaller amplitude. Despite varying in the generating mechanisms, aAPs have been reported in various brain cell types, including neurons, oligodendrocyte precursor cells (OPCs), and glioma cells. In this mini-review, we summarize the mechanisms and physiological functions of aAPs and outline their contributions to neurological diseases, particularly in glioma pathology. aAPs have been observed in mature brains, arising from mechanisms such as ectopic depolarizations and gap junction coupling, thereby supporting synaptic integration and network synchrony. It is also a signature of immature neurons in development. Subsets of NG2^+^ OPCs and immature oligodendrocyte-lineage cells exhibit state-, region-dependent excitability, ranging from subthreshold depolarizations to AP-like events, with potential roles in neuron–glial communication, ischemic vulnerability, and myelination. Accumulating human studies have demonstrated that glioma cells generate aAPs, while until recently their molecular profile was characterized by patch-seq. In IDH-mutant glioma, aAP cells exhibit a mixed GABAergic and OPC signature. At the leading edge (LE) of IDH-wild-type gliomas, aAPs are present in both adjacent non-tumor cells and glioblastoma cells (GBCs) across diverse GBC states, yet exhibit reduced proliferation and increased inflammatory signaling. In conclusion, aAPs are a recurrent but context-dependent electrophysiological feature observed in subsets of glioma cells, and may indicate an active role in network integration and active release. Dissecting the differential roles of aAP and no-aAP GBCs through targeted manipulations informed by transcriptomic results may reveal crucial mechanisms underlying multifaceted tumor-neuron crosstalk in glioma progression.

## 1 Introduction

**FIGURE 1:**
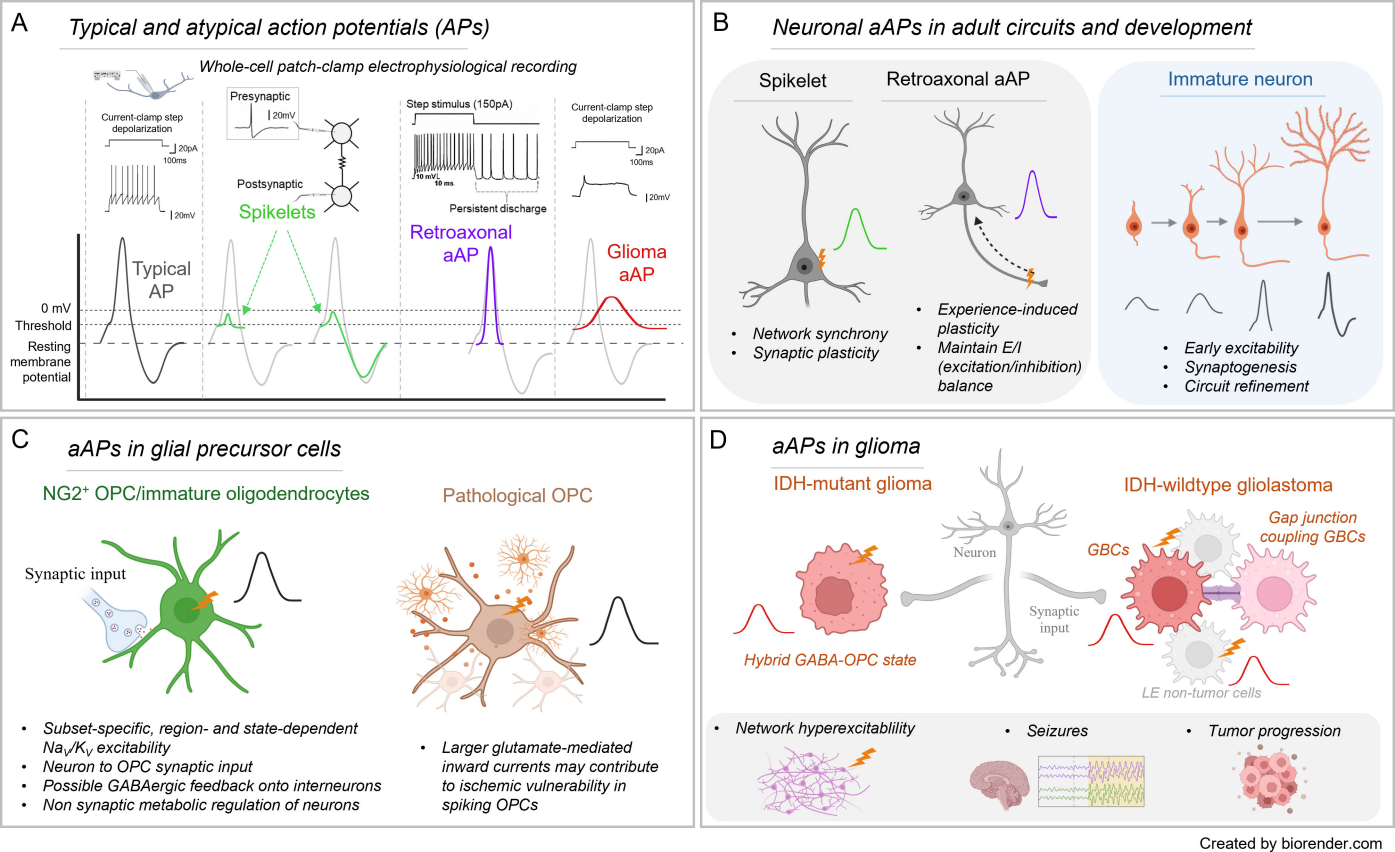
Atypical action potentials (aAPs) across physiological and pathological contexts. **(A)** Example waveforms of action potentials (APs) and atypical action potentials (aAPs) recorded using a somatic whole-cell patch-clamp configuration. Top, schematics illustrate recording configurations and stimulation protocols, including current-clamp step depolarization for APs [adopted from Ozsvár et al., 2024], paired recording for gap-junction filtered spikelets, step-pulse current injection for raAPs [adopted from Ozsvár et al., 2024], and near-threshold depolarization [adopted from Tong et al. (2026) for generating or identifying these events. Bottom, AP waveform (black trace) and aAP waveforms overlaid with the gray APs for comparison. aAPs include spikelets (green traces; left, spikelet waveform obtained from pyramidal cells; right, biphasic spikelet waveform from GABAergic fast-spiking neurons), retroaxonal APs (raAPs, magenta trace), and glioma-associated aAPs (red trace). **(B)** Neuronal aAPs in development and adult circuits. In mature neurons, somatic spikelets contribute to network synchrony, while raAPs support persistent activity during working memory tasks and maintain E/I balance. Immature neurons exhibit spikelet-like activity linked to early excitability, synaptogenesis, and circuit refinement. **(C)** aAPs in glial precursor cells. NG2-positive oligodendrocyte precursor cells and immature oligodendrocytes can exhibit subset-specific, region-dependent, and state-dependent Na_*V*_/K_*V*_-mediated excitability. These cells receive synaptic input from neurons and may participate in neuron-glia communication, GABAergic feedback, and non-synaptic metabolic regulation. Under pathological conditions, spiking OPC-like cells show enhanced glutamate-mediated inward currents and increased vulnerability to ischemic or excitotoxic stress. **(D)** aAPs in glioma. In IDH-mutant glioma, aAP-generating cells exhibit a hybrid GABAergic–OPC state with Na_*V*_/K_*V*_-dependent excitability. At the leading edge of IDH-wildtype glioblastoma, both tumor cells and neighboring non-tumor cells can generate aAPs spontaneously or in response to stimulation. Tumor cells also receive neuronal synaptic inputs and are coupled via gap junctions. Therefore, aAPs may serve as a glioma-mediated mechanism that promotes network hyperexcitability in the infiltrated zone, increases seizure susceptibility, and supports tumor progression. Created with BioRender.com.

The key features of nerve cells are being electrically active and their ability to generate action potentials (APs) (Bean, 2007). Typical APs are defined by (1) being recorded at the neuronal soma; (2) exhibiting an all-or-none property; (3) depending on voltage-gated sodium and potassium channels (Na_*V*_ and K_*V*_); (4) having an amplitude of approximately 50–100 mV; (5) initiating at the axon initial segment (AIS) (Manis, 2009). However, accumulating evidence since the 1990s indicates that both neurons and non-neuronal brain cells can generate either typical APs or spike-like, all-or-none small depolarizing events recorded at the soma. Here, we collectively refer to these spike-like events as atypical action potentials (aAPs). In this mini review, aAPs are defined as fast-depolarizing electrical spikes recorded from the cell body that violate either criterion (4) and/or (5). aAPs correspond to either spontaneously occurring events or the first evoked spike upon near-threshold positive current injections to the cell or its electrically coupled cells (Figure 1A). These aAPs are observed in the mature brain, during development, and in pathological contexts such as glioma, but with distinct features and functional implications (Curry et al., 2024; Fields, 2008; Stevens et al., 2024; Tong et al., 2026). The biological functions of aAPs are best characterized in neurons. (Michalikova et al., 2019), while much less is known in non-neuronal cells and in pathological conditions. Accumulating oncology studies reported that a subset of brain tumor cells can generate aAPs, shedding light on a new mechanistic insight into tumor cells’ active contribution to disease progression in cancer neuroscience research.

This minireview synthesizes emerging literature on the mechanistic diversity and functional implications of aAPs (summarized in Table 1). We cover contexts ranging from early brain development to adult network synchrony, as well as non-neuronal cells, specifically the recently identified aAP-generating glioma cells. We then further discuss the biological implications of aAPs in glioma cells, hypothesizing that their occurrence represents a neurodevelopmental-like feature, and its impact on the neuronal network and tumor microenvironment.

**TABLE 1:** Summary of atypical action potential reports discussed in this mini-review article.

Biological context	aAP features and mechanisms	Species	Condition	Cell type	Potential functional implications	References
Neurons and glial cells						
Adult brain	Spikelets •Attenuated axonal APs •Filtered APs through gap junction •Ephatic coupling	•Rats •Mice	Acute brain slices *in vitro.* •Cortex •Hippocampus	Glutamatergic pyramidal cells	Supports network synchronization and facilitating the generation of oscillations.	Galarreta and Hestrin, 2001a,b; Klueva et al., 2003; Mann-Metzer and Yarom, 1999; Szente et al., 2002
	Retroaxonal AP •Non-attenuated distal axonal APs •HCN1 dependent	•Rats •Mice	Acute brain slices *in vitro.* •Cortex •Hippocampus	Glutamatergic pyramidal cells	Supports persistent activity during working memory tasks, and may contribute to epileptiform-like network activity	Bähner et al., 2011; Chen et al., 2025; Dugladze et al., 2012; Elgueta et al., 2015; Thuault et al., 2013
	Retroaxonal AP •Non-attenuated distal axonal APs. •Persistent high-frequency firing upon induction •Modulation by axonal HCN channels	•Humans •Rats •Mice	Acute brain slices *in vitro* and *in vivo* patch-clamp. •Cortex •Hippocampus •Amygdala	GABAergic neurons •parvalbumin-expressing FS basket neurons •Neurogliaform cells	A compensatory internal mechanism suppressing local activity and avoiding overexcitation during persistent activation of pyramidal cells	Krook-Magnuson et al., 2011; Ozsvár et al., 2024; Rózsa et al., 2023; Sheffield et al., 2011
Neurodevelopment in cultures	Immature AP •Insufficient expression of Nav and Kv channels in the immature neurons. •Progressive increase in amplitude from single immature APs to full and multiple APs	•Humans	Human/ hESC/iPSC-derived cortical neuron *in vitro* culture	Immature pyramidal neurons	Functional neuronal cell differentiation linked to ion channel expression and synaptic integration	Bardy et al., 2016; Prè et al., 2014; Shi et al., 2012
Developmental brain	Immature AP •Insufficient expression of Nav and Kv channels in the immature neurons. •Increased ion channel density drives the maturation of AP properties. •Electrical coupling via gap junctions among immature neurons	•Rats •Mice	Acute brain slices in *vitro*. •Embryonic: Migratory intermediate zone and cortical plate. •Early postnatal: Neocortex.	Glutamatergic neurons	Neuronal maturation linked to ion channel expression and synaptic/network integration during development	Eustache and Gueritaud, 1995; Huguenard et al., 1988; Luhmann et al., 2000; McCormick and Prince, 1987; Pedroni et al., 2014; Picken Bahrey and Moody, 2003; Valiullina et al., 2016; Yu et al., 2012
Insect brain	Ectopic AP •Attenuated axonal APs recorded in vivo.	•Desert locust( Schistocerca gregaria) •Drosophila	Sharp electrode and patch-clamp *in vivo*. •Antennal lobe	•projection neurons (PNs) •Kenyon cells (KCs)	Soma may not be the primary site for integrating synaptic inputs and for information processing in insects	Gouwens and Wilson, 2009; Perez-Orive et al., 2002; Wilson et al., 2004
Non-neuronal cells	AP-like spikes and Spikelets. •Na_*V*_ and K_*V*_ dependency	•Rats •Mice	Acute brain slices *in vitro*. •Cerebellar white matter •Hippocampus •Cortex	•NG2^+^ oligodendrocyte precursor cells (OPCs) •SCN2A expressing immature oligodendrocytes	An electrical-active OPC subpopulation responding to environmental stimuli for further differentiation, with potential OPC-neuron feedback mechanisms	Berret et al., 2017; Chittajallu et al., 2004; Gould and Kim, 2021; Káradóttir et al., 2008; Zhang et al., 2021
Glioma cells						
Glioma (oligodendrogliomas and oligoastrocytoma) (pre-WHO 2021 classification)	Evoked single aAPs •Peaked ∼42 mV within 1.1 ms •Decay time 5.8 ms	Human	Acute brain slices from patient-derived tumor tissue located in the •Cerebral hemispheres	Oligodendrogliomas and Oligoastrocytoma cells from tumor center	•Neuronal mimicry in hijacking the brain network as developmental neurons to be further integrated in the circuit •aAP GBCs may be in transition state to become immature neuron •aAP supports an active release machinery of tumor cells in response to external stimulation and synaptic integration •Potential contribution to seizure activity in IDH-mutant glioma patients	Patt et al., 1996
Glioma (glioblastomas) (pre-WHO 2021 classification)	Evoked aAPs	Human	Acute brain slices from patient-derived tumor tissue. •Tumors were located supratentorially.	Glioblastomas cells		Labrakakis et al., 1997
Glioma (astrocytomas) (pre-WHO 2021 classification)	Evoked aAPs •∼52.5 mV amplitude •∼12 ms width	Human	Acute brain slice from pediatric patient-derived tumor tissue.	Astrocytomas cells		Bordey and Sontheimer, 1998
Glioma (IDH-mutant)	Evoked single aAP •Mixed GABAergic neuronal and OPC-like signatures.	•Human •Tumor transplant mouse model	Acute brain slice •Patient-derived tumor tissue •Genetically engineered glioma mouse model (Glast-targeted piggyBac IUE) •Patch-seq	IDH-mutant glioma cells		Curry et al., 2024
Glioma (IDH-wildtype)	Evoked single aAP	•Human •Tumor organoid transplant mouse model	Acute brain slice •Transplanted glioblastoma cells •Dorsal hippocampus	Primary and recurrent IDH-wildtype glioblastoma cells		Sun et al., 2025
Glioma (IDH-wildtype)	Evoked aAPs •∼27 mV amplitude •∼4.4 ms half-width •Some cells show spontaneous aAP-like transient events •Na_*V*_ and K_*V*_ dependency	•Human	Acute brain slice and organotypic slice culture •Tumor-infiltrated neocortex •Patch-seq	Tumor leading edge •Glioblastoma cells •Nearby non-tumor cells		Tong et al., 2026

The table summarizes experimental articles reporting aAPs across the distinct biological contexts, with a detailed report on glioma-related research articles. All electrophysiological recordings are performed using somatic whole-cell patch-clamp recordings unless specified otherwise.

## 2 Biophysical origins of distinct categories of atypical action potentials

In the central nervous system, aAPs have been observed in neurons using somatic patch-clamp recordings (Figures 1A, B). aAPs include at least three categories: (1) spikelets, which are attenuated APs; (2) retroaxonal aAPs (raAPs) that are non-attenuated APs arising from distal axonal compartments; and (3) aberrant APs that are small amplitude APs caused by insufficient Na_*V*_/K_*V*_ channel expression, observed mainly in immature neurons and glioma cells.

### 2.1 Adult mammalian brain

In the adult mammalian brain, spikelets are mainly reported in glutamatergic pyramidal neurons and fast-spiking (FS) GABAergic neurons of cortical structures, including the neocortex, hippocampus, and amygdala. In pyramidal neurons, somatic spikelet observation arises from distinct mechanisms, including electrically filtered AP via gap junctions, ephaptic coupling, and attenuated antidromic ectopic AP propagation [detailed mechanisms have been well-summarized and discussed in Michalikova et al. (2019)]. In GABAergic neurons, spikelets arise mainly from an AP filtered through the gap junction, manifesting a brief spikelet followed by slow hyperpolarization, yielding a synchronized, biphasic, and rhythmic membrane potential fluctuation between gap junction-connected FS neurons, facilitating the generation of oscillations (Galarreta and Hestrin, 2001b). Another study analyzing the correlation between spontaneous spiking patterns in gap junction-connected FS neurons revealed that 30% of the synchronized spikes are elicited by spikelets (Mann-Metzer and Yarom, 1999), and synchronous subthreshold inputs further promote synchronous spiking via mutual depolarization (Galarreta and Hestrin, 2001a,b).

The other type, raAPs, are ectopic spikes initiated in distal axons and propagate antidromically to the soma without attenuation. They exhibit a more negative initiation threshold but maintain peak amplitude and half-width comparable to those of typical APs. raAP are often triggered by sustained depolarizations, leading to a persistent barrage firing pattern. They have been reported both in glutamatergic pyramidal neurons (Bähner et al., 2011; Dugladze et al., 2012; Zhang et al., 2023) and GABAergic neurons, including the parvalbumin-expressing FS cells (Elgueta et al., 2015; Sheffield et al., 2011) and the neurogliaform cells (Krook-Magnuson et al., 2011; Ozsvár et al., 2024; Rózsa et al., 2023). Specific intrinsic channel mechanisms, such as the muscarinic acetylcholine receptor, the hyperpolarization-activated cation channel 1 (HCN1), and Ca^2+^ dependence, were proposed to be required for the generation of persistent raAPs (Chen et al., 2025; Egorov et al., 2002; Harris, 2008; Thuault et al., 2013). Persistent raAPs in pyramidal neurons are observed in the primate medial frontal cortex *in vivo* when sensory information must be retained for a short time period before choices are made during a delayed working memory task (Wang et al., 2013). The continuous raAPs within pyramidal cell microcircuits can maintain frontal neural excitation in the absence of “bottom-up” sensory inputs until decision making and have been proposed as a cellular mechanism for working memory (Goldman-Rakic, 1995). Elevated extracellular K^+^ level is sufficient to trigger persistent raAPs in amygdala pyramidal cells, which are observed alongside epileptiform-like network activity (Klueva et al., 2003). In GABAergic neurons, the persistent raAPs can be induced in vitro after several step-current injections, or by blocking synaptic inhibition or raising the extracellular K^+^ concentration, an *in vitro* model of epilepsy (Suzuki et al., 2014). Persistent raAPs produce prolonged and increased tonic inhibition onto nearby pyramidal cells and are therefore considered a compensatory mechanism to avoid overexcitation (Suzuki et al., 2014). Sporadic raAPs from neurogliaform cells can be directly measured in vivo, and their function may be to set up a brain-state-dependent cell output format, but this remains to be further investigated (Rózsa et al., 2023). In conclusion, aAPs in the adult mammalian brain represent the distinct initiation site and propagation mechanism, facilitating temporal precision in spike timing between neurons, and maintaining systemic excitation/inhibition balance.

### 2.2 Developmental roles of atypical spiking

During embryonic and early postnatal development, neurons undergo substantial changes in ion channel expression and membrane properties. Immature neurons display spikelet-like events — small-amplitude, partially overshooting depolarizations resembling attenuated APs — observed in developing cortical, hippocampal, and brainstem neurons (Eustache and Gueritaud, 1995; McCormick and Prince, 1987; Pedroni et al., 2014; Valiullina et al., 2016). Studies on human induced pluripotent stem cell (iPSC)-derived neurons show a progression from weak spike-like events to full, repetitive AP firing, coupled to increased Na^+^/K^+^ conductance, resting membrane hyperpolarization, synaptic maturation, and emerging network activity (Bardy et al., 2016; Prè et al., 2014; Shi et al., 2012). Similarly, studies in rodent brain slices show that early postnatal pyramidal neurons transition from high input resistance and slow, irregular spiking to faster, more reliable AP firing as Na^+^/K^+^ conductances increase (McCormick and Prince, 1987; Picken Bahrey and Moody, 2003). Early-born neurons such as Cajal–Retzius cells exhibit faster APs and higher Na^+^ current densities than immature pyramidal neurons, indicating a transient developmental hierarchy in which early-generated neurons dominate signal transmission (Luhmann et al., 2000).

Developing cortical neurons initially exhibit small Na_*V*_-dependent spikelets that precede full APs and are part of cell-autonomous activity that generates synchronized spontaneous retinal network activity (Galli and Maffei, 1988; Moody and Bosma, 2005; Zheng et al., 2006). Na_*V*_ blockade by bath application of tetrodotoxin (TTX) significantly reduces neuronal survival during development in an *in vitro* model (Voigt et al., 1997). Moreover, prenatal TTX infusion blocks layer-specific topographic map refinement of retinogeniculate axons in cats (Shatz and Stryker, 1988; Sretavan and Shatz, 1986). As neurons mature, increased Na_*V*_ current density and the emergence of persistent Na^+^ conductance convert spikelets into APs and support repetitive firing (Huguenard et al., 1988). Concurrently, the axon initial segment undergoes Na_*V*_1.2-to-Na_*V*_1.6 replacement and increased K_*V*_1/K_*V*_7 clustering, refining repolarization and stabilizing AP initiation, jointly stabilizing and lowering AP initiation threshold during maturation (Clark et al., 2009; Fréal et al., 2023; Garcia et al., 2025; Jenkins and Bender, 2025; Sánchez-Ponce et al., 2012; Susuki and Kuba, 2016). In addition, gap junction coupling among immature neurons propagates small depolarizations, promoting spikelet-like activity and synchronized firing in developing networks (Bennett and Zukin, 2004; Crodelle and McLaughlin, 2021; Elias and Kriegstein, 2008; Li et al., 2010, 2012; Montoro and Yuste, 2004; Peinado et al., 1993; Yu et al., 2012). Together, these processes delineate a coordinated sequence of biophysical and structural changes that transform immature neuronal depolarizations into APs.

Spikelet activity serves critical developmental functions by driving activity-dependent Ca^2 +^ influx via voltage-gated Ca^2 +^ channels (Ca_*V*_), regulating gene expression, and cytoskeletal remodeling (Arjun McKinney et al., 2022; Rosenberg and Spitzer, 2011). Spikelets promote axon pathfinding, dendritic arborization, synaptogenesis, and help synchronize developing assemblies before mature synaptic networks are established (Luhmann et al., 2016; Molnár et al., 2020; Nigi et al., 2025; Tezuka et al., 2022), representing a transitional mode of electrical signaling bridging passive membrane excitability to mature APs.

### 2.3 Non-mammalian brain

In insect brains, such as those of Drosophila and Locust, spikelets are detected in the projection neurons, such as mitral cells in the olfactory bulb or Kenyon cells in the mushroom body (Gouwens and Wilson, 2009; Perez-Orive et al., 2002; Wilson et al., 2004). A follow-up study supports the idea that the observed spikelets represent ectopic APs initiated at the proximal segment of the axon, which is distant from the soma (Gouwens and Wilson, 2009). These projection neurons in Drosophila have a unipolar morphology akin to that of peripheral neurons, meaning the soma is set far from the other compartments. This may imply that synaptic information processing occurs through a more direct dendritic-axon pathway, and the soma may not play a major role in integrating electrical signals.

## 3 Atypical spiking in glia precursor cells

CNS glial cells were considered non-excitable, but a subset of nerve/glial-antigen 2-expressing oligodendrocyte precursor cells (NG2^+^ OPCs) express Na_*V*_ and K_*V*_ and can generate subthreshold depolarization (Biase et al., 2010; Clarke et al., 2012; Zhang et al., 2021), or in some cases APs and aAPs upon current injections (Bakiri et al., 2009; Berret et al., 2017; Chittajallu et al., 2004; Fields, 2008; Gould and Kim, 2021; Káradóttir et al., 2008; Figure 1C). Chittajallu et al. (2004) reported that an aAP-generating subpopulation of NG2^+^ OPCs exists in the mouse cortex, but not in white matter. Káradóttir et al. (2008) identified two OPC subpopulations in rat white matter: a spiking subpopulation, expressing Na_*V*_ and K_*V*_, receiving synaptic inputs, and generating APs upon depolarization, and a non-spiking subpopulation lacking them. During simulated ischemia, the spiking OPCs showed increased glutamate-mediated synaptic currents, indicating vulnerability to excitotoxicity (Káradóttir et al., 2008). Another study by Berret et al. (2017) identified a subpopulation of pre-myelinating oligodendrocytes in the auditory brainstem that generates Na_*V*_1.2-driven APs and receives glutamatergic inputs. Na_*V*_1.2 knockdown altered oligodendrocyte morphology, impairing myelination and axon-oligodendroglial interactions (Berret et al., 2017). A follow-up study using patch-seq confirmed Na_*V*_1.2 is specifically associated with spiking OPCs at an OPC-to-pre-myelinating oligodendrocyte transition stage (Gould and Kim, 2021). These studies collectively suggest that oligodendrocyte-lineage excitability is state-, region-, and maturation-dependent. Whether it is a general feature across different regions, ages, and states remains an open question.

Functionally, OPC subpopulations actively receive neuronal inputs, which have been implicated in regulating their proliferation, differentiation, and myelin-forming potential (Bai et al., 2021; Bergles et al., 2000, 2010; Biase et al., 2010; Habermacher et al., 2019; Kukley et al., 2010; Lin and Bergles, 2004; Moura et al., 2022). In addition to neuron-to-OPC signaling, using high-resolution imaging and paired recordings, Zhang et al. (2021) reported NG2^+^ OPCs form GABAergic synaptic-like connections onto hippocampal interneurons, suggesting a feedback mechanism onto hippocampal inhibitory networks. Moreover, Fang et al. (2025) reported that an OPC subpopulation forms activity-dependent contacts with neuronal somata, promoting lysosome exocytosis and regulating neuronal metabolism, supporting OPC participation in activity-dependent local circuit regulation through both synaptic and non-synaptic mechanisms (Gobbo et al., 2024; Munyeshyaka and Fields, 2022; Zhang et al., 2021). Although the neurogenic potential of NG2^+^ OPCs remains debated, fate-mapping studies indicate that they are largely gliogenic (Livesey et al., 2016). The OPC maturation process is accompanied by a progressive decrease in Na_*V*_ and K_*V*_ currents and a loss of spiking ability, suggesting that spiking is a distinct feature of an actively differentiating or an active responding state in OPCs (Livesey et al., 2016).

## 4 Spikelet and AP activity in glioma

### 4.1 Glioma cells showing atypical AP

Early studies from the 1990s using human-derived tumor tissues found that glioma cells display immature neuron-like membrane activity, including high input resistance, presence of Na_*V*_/K_*V*_ currents, and occasional spike-like depolarizations, indicating intrinsic excitability in a subset of tumor cells (Bordey and Sontheimer, 1998; Labrakakis et al., 1997, 1998; Patt et al., 1996). Recent studies in human and tumoroid xenograft mouse models further elucidate the molecular makeup of aAP-generating tumor cells at high single-cell resolution using patch-seq (whole-cell patch-clamp combined with single-cell RNA sequencing). In human IDH-mutant glioma, a small fraction of cells, mainly glioma cells, exhibit mixed GABAergic-neuronal and OPC-like transcriptional signatures termed hybrid cells (HCs) that fire single Na_*V*_-dependent aAPs (Curry et al., 2024). In human IDH-wildtype glioblastoma, a substantial fraction of leading-edge glioblastoma cells (LE GBCs) across heterogeneous cellular states generates small, Na_*V*_/K_*V*_-dependent aAPs with kinetics similar to adjacent non-tumor cells (Tong et al., 2026). Nearby non-tumor cells with neuronal-like signatures also exhibit similar aAPs, suggesting shared electrophysiological features within the tumor microenvironment (Tong et al., 2026). aAP-like responses are also evoked in mouse models bearing human-derived glioblastoma tumoroid xenografts (Sun et al., 2025). Together, aAPs represent a recurrent but context-dependent electrophysiological feature in subsets of glioma cells across tumor subtypes and model systems.

### 4.2 aAP in glioma cells: implication and therapeutic perspective

Glioblastoma develops within a highly interconnected neural ecosystem, where malignant cells establish reciprocal interactions with neurons, glia, and the vascular niche (Leake, 2023; Monje, 2025; Monje and Winkler, 2025; Stevens et al., 2024; Venkataramani et al., 2025; Figure 1D). Accumulating evidence indicates that subsets of glioma cells display neuronal-like electrophysiological properties, integrating into pre-existing neural circuits through chemical synapses (Barron et al., 2025; Sun et al., 2025; Tetzlaff et al., 2025; Venkataramani et al., 2019; Venkatesh et al., 2019). Excitatory glutamatergic synapses from neurons represent a primary mode of tumor–neuron communication, driving depolarization, Ca^2 +^ influx, and activity-dependent tumor growth (Venkataramani et al., 2019; Venkatesh et al., 2019), while inhibitory GABAergic inputs modulate glioma cell excitability depending on intracellular chloride gradients and receptor composition (Barron et al., 2025). Additional neuromodulatory inputs, including cholinergic signaling, further diversify the synaptic regulation of tumor cells and further complicate neuron–glioma interactions (Sun et al., 2025; Tetzlaff et al., 2025). Beyond chemical synapses, glioma cells are electrically coupled through gap junctions, forming multicellular networks that support the propagation of depolarizing signals across the tumor mass (Hausmann et al., 2023; Osswald et al., 2015; Venkataramani et al., 2022). In this context, aAP may represent glioma cells’ functional integration into neural network dynamics and may underline pathological manifestations such as seizures in IDH-mutant glioma patients (Curry et al., 2024).

Gliomas also shape the microenvironment by secreting non-synaptic glutamate in an activity-dependent manner, leading to aberrant synaptogenesis, hyperexcitability, excitotoxicity, and neuronal degeneration (Buckingham et al., 2011; Campbell et al., 2012; Yu et al., 2020). Sustained hyperexcitability can depolarize neuronal resting membrane potentials and, under excessive stimulation such as epileptic activity, lead to axonal degeneration and block AP firing (Ying Cao, 2024). In this context, aAP from tumor cells may further reinforce local network hyperexcitability through activity-dependent glutamate release, though the coupling mechanism remains unclear. Conversely, neuronal activity can promote glioma progression through paracrine signaling (Taylor et al., 2023; Venkatesh et al., 2015, 2017).

Given the genetic and cellular heterogeneity of glioma (Neftel et al., 2019), the occurrence and implications of aAP may depend on tumor subtype and cellular context. In human IDH-mutant glioma, lower representation of spiking HCs correlates with poorer patient survival, suggesting that the presence of spiking HCs marks a less aggressive phenotype (Curry et al., 2024). As spiking HCs show mixed GABAergic and OPC signatures, comparing their properties to those of spiking OPCs and developing GABAergic neurons may help elucidate their function. By contrast, in human IDH-wildtype glioblastoma, aAPs are observed across heterogeneous tumor cell states without a clear GABA-OPC-like transcriptional signature, and LE aAP-GBCs show reduced proliferation-related signaling and enriched mesenchymal transitioning pathways relative to LE no-aAP GBCs (Tong et al., 2026). We postulate aAPs serve tumor type-, location-, and function-specific roles. aAP-GBCs at the LE may occupy a hybrid stage with immature-neuron features, exploiting migration routes shared with immature neurons and stem cells. Region- and subtype-specific *in vivo* manipulations are needed to elucidate aAP contributions across tumor subtypes further.

The recognition of aAP and spikelet in gliomas opens avenues for potential therapeutic strategies. Ion channel blockers, gap junction inhibitors, or modulators of ephaptic interactions could mitigate tumor-induced circuit dysfunction (Wick et al., 2018). In addition to previously identified Na_*V*_/K_*V*_ blockers to suppress tumor activity and progression, several subtype-specific targets identified by patch-seq in (Tong et al., 2026) provide new strategies targeting LE GBCs: (1). Ablation of general increased CCND2 expression on LE GBCs (a gene required for cell division); (2). Ablation of abundantly expressed Na_*V*_1.3 in aAP-LE GBCs; (3). Ablation of enriched Ca_*V*_1.2 expression in no-aAP LE GBCs. These approaches remain preliminary and technically challenging, with limitations including viral delivery, off-target effects, and tumor-subtype specificity. High-resolution electrocorticography mapping of abnormal spiking may improve early glioma detection and monitoring, and transcranial magnetic stimulation or deep-brain stimulation might normalize local excitability in glioma patients with seizures. Computational models integrating developmental, physiological, and pathological data will be essential for predicting the impact of targeted interventions.

## 5 Conclusion and future directions

Spikelet and aAP are not epiphenomena but are mechanistically and functionally significant across brain development and disease. From shaping early circuits to perturbing adult networks in glioma, these electrical events exemplify the brain’s dynamic excitability landscape. Continued exploration of their mechanisms and consequences will not only deepen our understanding of brain function but also unlock new possibilities for clinical translation in neuro-oncology.
